# Induction of endotoxin tolerance in murine monocyte and macrophage cell populations – optimal LPS dose and compartment-specific reversal by β-glucan†

**DOI:** 10.1039/d4fo05223d

**Published:** 2025-02-07

**Authors:** Bart G. J. Moerings, Coen Govers, Jeroen van Bergenhenegouwen, Jurriaan J. Mes, Miriam van Dijk, Renger F. Witkamp, Klaske van Norren, Suzanne Abbring

**Affiliations:** a Division of Human Nutrition and Health, Wageningen University & Research Wageningen The Netherlands suzanne.abbring@wur.nl; b Wageningen Food and Biobased Research, Wageningen University & Research Wageningen The Netherlands; c Cell Biology and Immunology Group, Wageningen University & Research Wageningen The Netherlands; d Danone Nutricia Research Utrecht The Netherlands

## Abstract

Beta-glucans, naturally present in foods like wheat, mushrooms, and yeast, have shown potential in reversing immunosuppression. However, the existing evidence solely relies on *ex vivo* studies assessing direct effects of β-glucans on macrophages. To investigate whether such effects also occur after their oral administration, this study first systematically examined the immunosuppressive effects of LPS in mice. Subsequently, we assessed the ability of yeast-derived whole β-glucan particles (yWGP), administered through the diet, to counteract LPS-induced immunological tolerance. Immunosuppression following intraperitoneal administration of 20, 200, or 2000 μg kg^−1^ LPS was demonstrated by reduced TNF-α and IL-6 release upon *ex vivo* LPS stimulation of immune cells harvested from the peritoneal fluid, spleen, and bone marrow. Immunosuppression in blood was detected only after 200 and 2000 μg kg^−1^ LPS. LPS tolerance extended to heterologous stimuli (PAM3Cys, heat-killed *Pseudomonas aeruginosa*), indicating cross-tolerance. Due to animal discomfort at 2000 μg kg^−1^ LPS, as evidenced by a significantly enhanced clinical severity score, a dose of 200 μg kg^−1^ LPS was selected for the follow-up trial. In this experiment, mice fed a yWGP-supplemented diet for two weeks prior to LPS administration showed effective reversal of LPS tolerance, reflected by restored TNF-α levels in peritoneal cells but not in other monocyte- and macrophage-containing cell populations. Together, these studies demonstrate that peritoneal administration of 200 μg kg^−1^ LPS induced *ex vivo* LPS tolerance in all immunological organs studied, without significantly compromising animal welfare. The selective efficacy of dietary β-glucans to counteract immunosuppression, which is often observed in vulnerable and immunocompromised patient populations, warrants further clinical evaluation.

## Abstract 2

Dietary β-glucan has the potential to selectively counteract LPS-induced immunosuppression.

## Graphical abstract

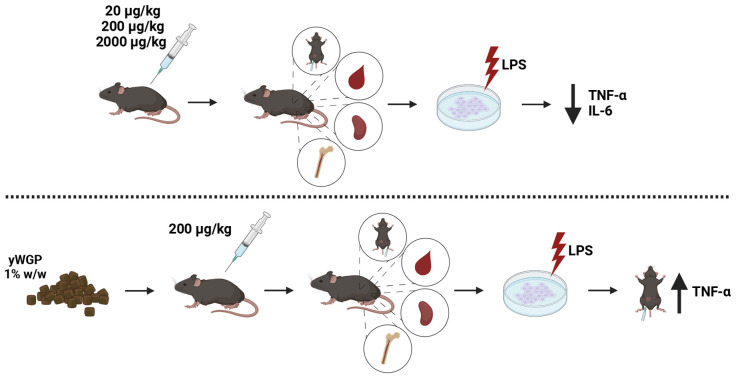

## 1. Introduction

The global population is aging rapidly, and projections indicate that by 2050 the number of people over 60 years of age will reach 2 billion.^1^ Aging is accompanied by changes in the immune system that lead to a gradual decline in immune function which is referred to as immunosenescence.^2,3^ A hallmark of immunosenesence is chronic low-grade inflammation, often referred to as inflammaging, to which the body activates a compensatory immunosuppressive mechanism, to protect tissues from excessive inflammatory injuries.^4^ This, however, also limits the efficacy of the immune system to respond, resulting in reduced effectiveness of vaccination^5^ and increased risk of infections.^6^

Particularly, innate immune cells, such as monocytes and macrophages, play an important role in the increased susceptibility to opportunistic infections resulting from immunosuppression.^7^ Immunosuppression is often initiated by a primary activation, which triggers a reduced ability of monocytes and macrophages to respond to a secondary microbial challenge. Macrophages show a decreased production of tumor necrosis factor alpha (TNF-α), interleukin (IL)-12, and IL-6 upon secondary stimulation, while exhibiting increased secretion of anti-inflammatory cytokines such as IL-10 compared to naïve cells that did not receive a primary stimulus.^8^ This hyporesponsive or refractory state is also known as endotoxin tolerance.^9–11^ A similar phenomenon occurs in cross-tolerance, where exposure to a specific ligand reduces the inflammatory response to other ligands, albeit to a lesser extent.^8,12^

Reducing the consequences of immunosuppression, whether or not specific to a primary challenge, holds the potential to reduce the risk of opportunistic infections, which could be beneficial to overall health and quality of life of older individuals. Immune-supportive nutritional components, such as β-glucans, may present a readily available opportunity to enhance immune fitness.^13^ Beta-glucans are cost-effective, well tolerated, and can be taken orally, making them promising candidates to boost immune fitness.^14^*In vitro* studies have demonstrated that stimulating tolerized macrophages with β-glucans can partially reverse lipopolysaccharide (LPS)-induced tolerance,^15^ and *ex vivo* treatment with β-glucan has been shown to restore cytokine production capacity in monocytes obtained from volunteers with experimental endotoxemia.^16^ However, these findings originate solely from *ex vivo* studies where there is direct exposure of β-glucan to monocytes, whereas the *in vivo* situation involving oral ingestion is more complex due to effects of the matrix, digestive and fermentative gastrointestinal tract activity, intestinal absorption, and peripheral distribution. The latter is demonstrated by studies following the fate of fluorescently or radiolabeled dietary fibers which could be detected in spleen, bone marrow, and urine after oral intake.^17,18^ To prove the effectiveness of orally ingested β-glucans in counteracting immunosuppression, it is crucial to obtain supportive evidence in preclinical models.

Therefore, we first established a model of *ex vivo* tolerance by testing different *in vivo* LPS doses aiming to elicit a reduced immune response upon a secondary *ex vivo* challenge. Next, as main objective, we investigated whether dietary intake of yeast-derived whole β-glucan particle (yWGP) can enhance immune fitness by counteracting LPS-induced immunological tolerance.

## 2. Materials and methods

### 2.1 Animals

Four-week-old male C57BL/6J mice were purchased from Envigo (Horst, The Netherlands) and arrived at the animal facilities in the desired group size to prevent re-randomization and minimize the risk of fighting. Housing was in conventional type II open cages with wood wool bedding, a tunnel, and a gnawing stick, under a 12 h light/dark cycle in humidity- (56.7 ± 4.2%, mean ± SD) and temperature (21.2 ± 0.3 °C)-controlled conditions with unrestricted access to food and water. Upon arrival, cages were randomly assigned to the control and experimental groups and mice were habituated to laboratory conditions and the AIN-93M control diet (Ssniff Spezialdiäten GmbH, Soest, Germany) for 2 weeks prior to the start of the study. This research was carried out with ethical approval from the national competent authority (CCD, Centrale Commissie Dierproeven) including a positive advice from the local animal ethics committee (Wageningen University, The Netherlands). All animal procedures were sanctioned by the Animal Welfare Body and adhered to the principles of good laboratory animal care, ensuring complete adherence to the European Directive 2010/63/EU governing the use of animals for scientific purposes.

### 2.2 Experimental design

**Fig. 1:**
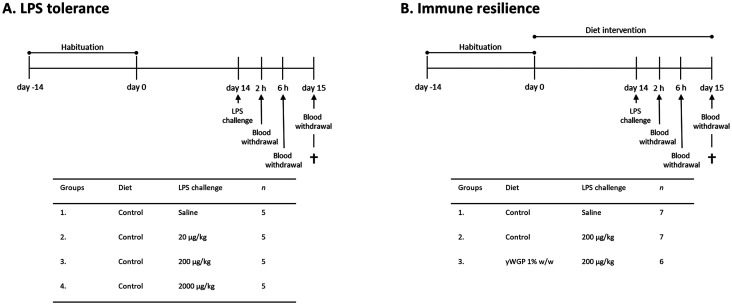
Schematic overview of the study designs. (A) Experimental timeline of the LPS tolerance study. (B) Experimental timeline of the immune resilience study.

In the first study (Fig. 1A), all animals (*n* = 5 per group) remained on the AIN-93M control diet for an additional 2 weeks following the 2 weeks habituation period. After these two weeks, on experimental day 14, mice were intraperitoneally (i.p.) injected with LPS (derived from *Escherichia coli* serotype O111:B4; Sigma-Aldrich, Zwijndrecht, The Netherlands) at three different doses: 20, 200, and 2000 μg kg^−1^, or a similar volume (0.2 mL) of PBS. Two and six hours after LPS administration, a blood sample was taken *via* tail incision to assess circulatory cytokine levels. At these timepoints, the clinical condition of the animals was also evaluated using a clinical severity score (CSS) to monitor the degree and severity of LPS-induced systemic inflammation.^19^ Symptoms included in the CSS were appearance of piloerection, level of consciousness, activity, response to stimulus, eye opening, respiration rate, and respiration quality. Each of these symptoms was scored between 0 and 4. A CSS of >14, or an increase of more than 3 points on any of the variables was defined as humane endpoint. Heat mats were provided to alleviate the distress. Biotechnicians scoring the health of the animals were blinded to treatment. Twenty-four hours after LPS administration, the CSS was assessed again. Mice were subsequently anesthetized with isoflurane followed by blood collection *via* orbital bleeding. Immediately thereafter, mice were euthanized by cervical dislocation, and organs were collected for *ex vivo* analysis of LPS-induced immunological tolerance.

In a follow-up study (Fig. 1B), the effect of yWGP on LPS tolerance was assessed. The experimental design is comparable to that of the first study. Shorty, mice in the control groups (*n* = 7 per group) remained on the AIN-93M control diet for 2 weeks after the habituation period. Mice in the experimental group (*n* = 6 per group) switched to a 1% w/w yWGP (Invivogen, Toulouse, France)-supplemented diet (Table S1,† Ssniff Spezialdiäten GmbH) in which yWGP was added at the expense of cellulose (12.7 g kg^−1^ dry matter, corrected for fiber purity), resulting in an average daily food intake of 3.3 g, as previously described.^20^ Two weeks later, mice were challenged i.p. with 200 μg kg^−1^ LPS or PBS (as a control). Twenty-four hours after the LPS challenge, mice were euthanized, and organs were collected to assess immune resilience *ex vivo*. The physicochemical characteristics of yWGP, such as solubility, protein content, molecular weight distribution, branching and linkages, monosaccharide composition, total saccharide content (*i.e.*, purity), and LPS/LTA contamination levels can be found in Table S2.†

### 2.3 Analysis of LPS-induced tolerance *ex vivo*

Various monocyte- and macrophage-containing cell populations were isolated and stimulated *ex vivo* to determine cytokine production as a measure of LPS-induced tolerance.

Following euthanasia of mice, peritoneal cells were isolated by injecting 10 mL of ice-cold PBS + 2 mM EDTA (ThermoFisher Scientific, Waltham, MA, USA) into the peritoneal cavity. After gently massaging the abdomen, the fluid containing peritoneal cells was either collected by making a small incision in the skin (study 1) or by using a 10 mL syringe and a 24G needle (study 2). The cell suspension was subsequently passed through a 70 μm cell strainer (Falcon, Corning Incorporated, Corning, NY, USA), centrifuged (1400*g*, 7 min), and incubated on ice with 1 mL lysis buffer (1.5 M NH_4_Cl, 0.1 M NaHCO_3_, and 10 mM EDTA [all from Merck, Darmstadt, Germany] in demineralized water, filter-sterilized) to remove red blood cells. Peritoneal cells were then resuspended in RPMI 1640 – Glutamax – HEPES medium supplemented with 10% FBS, 1% MEM non-essential amino acids, 1% sodium pyruvate, and 1% penicillin/streptomycin (all from Gibco, Bleiswijk, The Netherlands) and added to a 96-well tissue culture plate (2 × 10^5^ cells per well; Costar, Corning Incorporated). Cells were treated with 1 μg mL^−1^ LPS for 4 h at 37 °C, 5% CO_2_. After 4 h, plates were centrifuged, and supernatants were collected and stored at −20 °C for further analysis.

To isolate splenocytes, spleens were collected and homogenized using a syringe and a 70 μm cell strainer. These homogenized single cell suspensions were then incubated with lysis buffer to eliminate red blood cells before being resuspended in culture medium. Subsequently, splenocytes (5 × 10^5^ cells per well) were cultured in 96-well tissue culture plates and stimulated with 1 μg mL^−1^ LPS, 10 μg mL^−1^ PAM3Cys (EMC Microcollections, Tübingen, Germany), or 1 × 10^7^ cells per mL heat-killed *Pseudomonas aeruginosa* (HK-PA; InvivoGen, San Diego, CA, USA) for 24 h at 37 °C, 5% CO_2_. After 24 h, plates were centrifuged and supernatants were collected and stored at −20 °C for further analysis.

For bone marrow-derived cells, hind legs were extracted at the hip joint, and bone marrow was flushed from both femurs and tibiae using a needle and syringe. The resulting bone marrow cell suspension was filtered through a 70 μm cell strainer, centrifuged (1400*g*, 7 min) and treated with lysis buffer to eliminate red blood cells. Subsequently, cells were resuspended in culture medium, plated in 24-well tissue culture-treated plates (5 × 10^5^ cells per well), and exposed to 1 μg mL^−1^ LPS, 10 μg mL^−1^ PAM3Cys, or 1 × 10^7^ cells per mL HK-PA for 24 h at 37 °C and 5% CO_2_. After 24 h, supernatants were harvested and stored at −20 °C for further analysis. Additionally, bone marrow cells were cultured in 24-well tissue culture-treated plates (5 × 10^5^ cells per well) with 50 ng mL^−1^ macrophage colony-stimulating factor (M-CSF; R&D Systems, Minneapolis, MN, USA) to differentiate into bone marrow-derived macrophages (BMDMs) over 1 week. On day 4, the medium was refreshed with new culture medium containing 50 ng mL^−1^ M-CSF. On day 7, cells were stimulated with LPS, PAM3Cys, or HK-PA for 24 h at 37 °C and 5% CO_2_. Following this incubation, supernatants were collected and stored at −20 °C for further analysis.

### 2.4 Whole blood stimulation *ex vivo*

Blood was obtained *via* orbital bleeding and collected in lithium heparin tubes. For *ex vivo* stimulation, lithium heparin-anticoagulated blood (100 μL) was stimulated directly in the lithium heparin tubes 1 : 1 with 1 μg mL^−1^ LPS or plain RPMI 1640 medium (Corning) for 6 h at 37 °C, 5% CO_2_. Samples were subsequently centrifuged for 10 min at 2000*g*. Plasma was aliquoted and stored at −80 °C until further analysis.

### 2.5 Cytokine measurements

Lithium heparin-anticoagulated blood was collected 2, 6, and 24 h after LPS administration to assess circulating cytokine levels. Blood samples were centrifuged at 2000*g* for 10 min and plasma was stored at −80 °C until cytokine analysis. Plasma concentrations of TNF-α, IL-6, monocyte chemoattractant protein-1 (MCP-1), IL-1β, and IL-10 were determined using a LEGENDplex (BioLegend, San Diego, CA, USA) according to the manufacturer's instructions. The production of TNF-α and IL-6 in supernatants of *ex vivo* stimulations was determined by means of ELISA (BioLegend, San Diego, CA, USA) according to manufacturer's protocol.

### 2.6 Statistical analysis

Data are presented as mean ± standard error of the mean (SEM), except for the CSS values, which are presented as medians with interquartile ranges. Statistical differences compared to the control group were determined using one-way ANOVA followed by Dunnett's multiple comparisons test. In cases where the data did not follow a normal distribution (as assessed by the Kolmogorov–Smirnov test), log transformation was applied. If log transformation did not lead to normality, the non-parametric Kruskal–Wallis test followed by Dunn's multiple comparisons test was used. The Kruskal–Wallis test was also used to analyze CSS values. Plasma cytokine levels were analyzed using two-way repeated measures ANOVA followed by Dunnett's or Bonferroni's multiple comparisons test. All statistical analyses were performed using GraphPad Prism software (version 9.3.1; GraphPad Software, San Diego, CA, USA) and results were considered statistically significant when *P* < 0.05.

## 3. Results

### 3.1 Dose-dependent increase in systemic cytokine concentrations following *in vivo* LPS administration

**Fig. 2:**
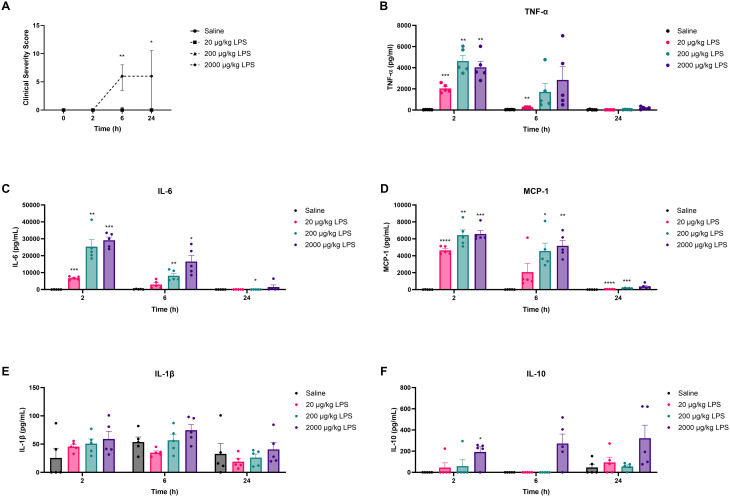
LPS-induced changes in health status and plasma cytokine concentrations in mice. Mice were i.p. injected with different doses of LPS (20, 200, and 2000 μg kg^−1^) or a similar volume (0.2 mL) of PBS. The clinical severity score (CSS) was assessed over the course of 24 h (A). TNF-α (B), IL-6 (C), MCP-1 (D), IL-1β (E), and IL-10 (F) concentrations were measured in plasma 2, 6, and 24 h after i.p. LPS challenge. Data are presented as medians with interquartile ranges for the CSS and as mean ± SEM for the plasma cytokine concentrations, *n* = 5 per group. *P < 0.05, **P < 0.01, ***P < 0.001, ****P < 0.0001, as analyzed with Kruskal–Wallis test for non-parametric data followed by Dunn's multiple comparisons test (A) or two-way repeated measures ANOVA followed by Dunnett's multiple comparisons test (B–F).

To establish the model and determine the dose of LPS that would elicit *ex vivo* tolerance, mice were i.p. administered with 20, 200, or 2000 μg kg^−1^ LPS. Two, six, and twenty-four hours after LPS administration, clinical severity scores (CSS) and inflammatory cytokine levels in plasma were assessed to determine the extent of LPS-induced systemic inflammation. As shown in Fig. 2A, only mice exposed to 2000 μg kg^−1^ LPS displayed significantly increased CSS compared to control animals exposed to saline. Maximum CSS values in these animals were reached 6 h after LPS administration (Fig. 2A). As expected, all CSS scores remained well below the humane endpoint (a clinical severity score > 14).

Plasma cytokine concentrations generally increased dose-dependently upon LPS administration as illustrated in Fig. 2B–F. At 2 h post-administration, all three LPS doses significantly increased TNF-α (Fig. 2B), IL-6 (Fig. 2C), and MCP-1 (Fig. 2D) levels compared to control. After 6 h, IL-6 and MCP-1 concentrations showed a significant increase only at doses of 200 and 2000 μg kg^−1^ LPS, whereas for TNF-α, a significant increase was only observed after the administration of 20 μg kg^−1^ LPS (Fig. 2B–D). Over time, TNF-α, IL-6, and MCP-1 levels gradually decreased and nearly returned to baseline after 24 h (Fig. 2B–D). LPS administration did not affect IL-1β levels at any of the measured timepoints (Fig. 2E). To investigate potential compensatory mechanisms, plasma IL-10 concentrations were also measured. LPS administration of 2000 μg kg^−1^ significantly increased IL-10 levels at 2 h, whereas 20 and 200 μg kg^−1^ caused no significant alteration in plasma IL-10 levels compared to control (Fig. 2F). At 6 and 24 h, none of the LPS groups differed significantly from control (Fig. 2F).

### 3.2 *In vivo* LPS administration diminishes pro-inflammatory cytokine production by murine monocyte- and macrophage-containing cell populations in response to an *ex vivo* LPS challenge

**Fig. 3:**
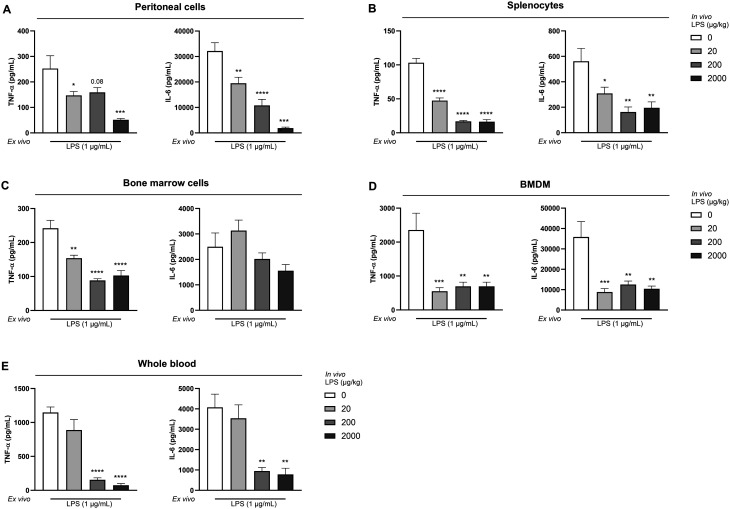
*In vivo* LPS exposure reduced TNF-α and IL-6 production in various monocyte- and macrophage-containing cell populations after *ex vivo* LPS challenge. TNF-α and IL-6 concentrations were measured in supernatant of *ex vivo* LPS-stimulated peritoneal cells (A), splenocytes (B), bone marrow cells (C), BMDMs (D), and whole blood cultures (E) from mice i.p. injected with different doses of LPS (20, 200, and 2000 μg kg^−1^) or PBS (as a control). Data are presented as mean ± SEM, *n* = 5 per group. *P < 0.05, **P < 0.01, ***P < 0.001, ****P < 0.0001, as analyzed with one-way ANOVA followed by Dunnett's multiple comparisons test.

In order to investigate LPS tolerance *ex vivo*, various cell populations containing monocytes and macrophages were isolated 24 h after i.p. LPS administration, re-exposed to LPS *ex vivo* and tested for TNFα and IL-6 secretion. As shown in Fig. 3A, peritoneal cells from mice injected with LPS exhibited reduced secretion of TNF-α and IL-6 following a secondary *ex vivo* LPS stimulation, compared to cells from mice injected with saline. Similar effects were observed in splenocytes (Fig. 3B), bone marrow cells (Fig. 3C), BMDMs (Fig. 3D), and whole blood cultures (Fig. 3E). Exceptions were IL-6 production by bone marrow cells from LPS-exposed animals, which was not significantly reduced compared to control animals upon a secondary LPS stimulation *ex vivo*. Next to this, TNF-α and IL-6 secretion levels were also not reduced upon secondary *ex vivo* LPS stimulation of whole blood samples from mice challenged with 20 μg kg^−1^ LPS (Fig. 3C). *In vivo* administration of 200 and 2000 μg kg^−1^ LPS resulted in the strongest tolerance response assessed *ex vivo*. At these doses, significant differences between PBS- and LPS-administered mice were observed in all cell compartments, in contrast to the 20 μg kg^−1^ LPS dose (Fig. 3A–E).

### 3.3 Murine monocyte- and macrophage-containing cell populations demonstrate cross-tolerance to PAM3Cys and HK-PA following *in vivo* LPS exposure

**Fig. 4:**
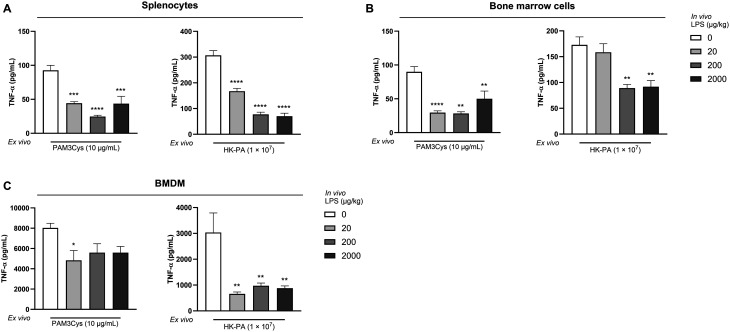
Systemic dosing with LPS *in vivo* resulted in cross-tolerance to PAM3Cys and HK-PA *ex vivo*. The release of TNF-α by splenocytes (A), bone marrow cells (B), and BMDMs (C) from mice *in vivo* exposed to LPS (20, 200, and 2000 μg kg^−1^) or PBS was measured in supernatant collected 24 h after *ex vivo* PAM3Cys and HK-PA stimulation. Data are presented as mean ± SEM, *n* = 5 per group. *P < 0.05, **P < 0.01, ***P < 0.001, ****P < 0.0001, as analyzed with one-way ANOVA followed by Dunnett's multiple comparisons test.

Having established that *in vivo* LPS administration induces *ex vivo* LPS tolerance, we proceeded to assess whether splenocytes and bone marrow-derived cells from LPS-exposed animals also showed cross-tolerance to heterologous *ex vivo* stimuli. Cross-tolerance could not be assessed in peritoneal cells and blood cultures due to a limited number of cells. Similar to LPS tolerance, decreased levels of TNF-α and IL-6 were observed in *ex vivo* PAM3Cys- and HK-PA-stimulated splenocytes obtained 24 h after i.p. LPS administration (Fig. 4A and Fig. S1A†). This hyporesponsiveness was also observed for TNF-α production by bone marrow cells after stimulation with both PAM3Cys and HK-PA (Fig. 4B). The secretion of IL-6 by bone marrow cells was not altered (Fig. S1B†). In addition, BMDMs cultured from LPS-exposed mice displayed decreased TNF-α and IL-6 production in response to HK-PA stimulation (Fig. 4C and Fig. S1C†). Upon PAM3Cys stimulation, only the TNF-α production was significantly reduced in animals exposed to 20 μg kg^−1^ LPS, whereas IL-6 production was not affected (Fig. 4C and Fig. S1C†).

### 3.4 Intake of yWGP did not affect plasma cytokine concentrations following *in vivo* LPS administration

**Fig. 5:**
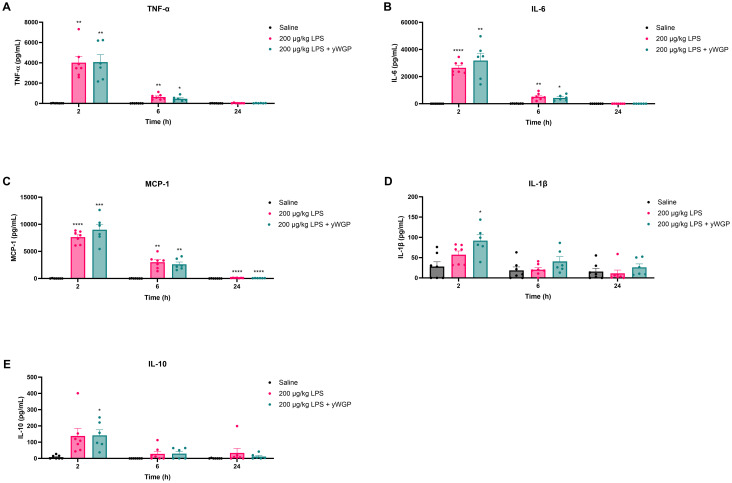
No effect of yWGP on plasma cytokine levels upon an i.p. LPS challenge. TNF-α (A), IL-6 (B), MCP-1 (C), IL-1β (D), and IL-10 (E) concentrations were measured in plasma from control, tolerized and 1% w/w yWGP-exposed animals 2, 6, and 24 h after i.p. LPS challenge (200 μg kg^−1^). Data are presented as mean ± SEM, *n* = 7 for control and LPS-tolerized animals and *n* = 6 for yWGP-exposed animals. *P < 0.05, **P < 0.01, ***P < 0.001, ****P < 0.0001, as analyzed with two-way repeated measures ANOVA followed by Bonferroni's multiple comparisons test.

Based on the previous, we continued with the model using 200 μg kg^−1^ LPS i.p. administration since that effectively induced *ex vivo* tolerance while minimizing animal discomfort. To investigate the potential of yWGP to reduce LPS-induced tolerance, mice were fed a 1% w/w yWGP-supplemented diet for two weeks. This yWGP concentration was selected based on our previous findings, demonstrating that oral intake of 1% w/w yWGP enhanced innate immune responsiveness *in vivo*.^20^ Mice exposed to 200 μg kg^−1^ LPS, whether or not in combination with the yWGP-supplemented diet, showed no impaired welfare. Similar to the control animals, they had a CSS of zero (data not shown). Comparable to the first study, administration of 200 μg kg^−1^ LPS resulted in elevated plasma levels of TNF-α, IL-6, and MCP-1 compared to saline administration, whereas this did not significantly affect IL-1β and IL-10 concentrations (Fig. 5A–E). Highest pro-inflammatory cytokine levels were again observed 2 h after LPS administration (Fig. 5A–D). Exposure to yWGP did not alter LPS-induced cytokine levels compared to LPS control animals (Fig. 5A–E).

### 3.5 yWGP counteracts LPS-induced tolerance in peritoneal cells

**Fig. 6:**
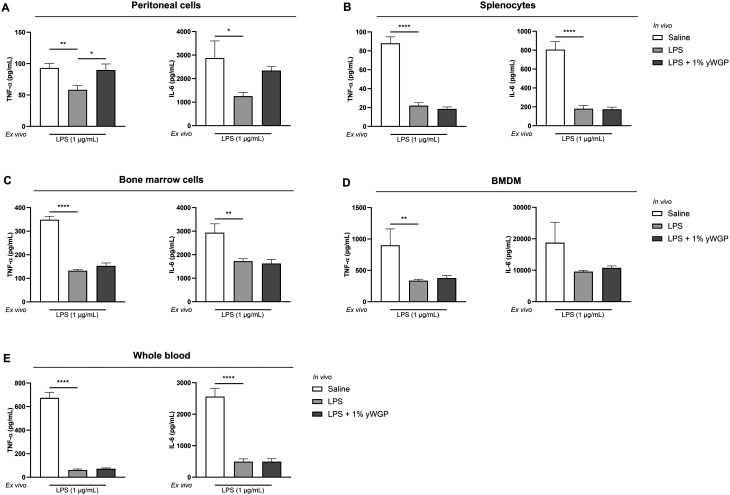
yWGP counteracted LPS-induced tolerance in peritoneal cells. TNF-α and IL-6 release by peritoneal cells (A), splenocytes (B), bone marrow cells (C), BMDMs (D), and whole blood cultures (E) from control, LPS-tolerized, and 1% w/w yWGP-exposed animals after *ex vivo* LPS stimulation. Data are presented as mean ± SEM, *n* = 7 for control and LPS-tolerized animals and *n* = 6 for yWGP-exposed animals. *P < 0.05, **P < 0.01, ****P < 0.0001, as analyzed with one-way ANOVA followed by Dunnett's multiple comparisons test.

To investigate whether oral yWGP administration counteracts LPS-induced tolerance, multiple cell populations containing monocytes and macrophages from control mice and mice exposed to 1% w/w yWGP (both groups i.p. challenged with 200 μg kg^−1^ LPS) were stimulated *ex vivo* with LPS. As shown in Fig. 6, peritoneal cells, splenocytes, bone marrow cells, and whole blood cultures from tolerized mice showed significantly reduced secretion of TNF-α and IL-6 compared to naïve control mice following a secondary *ex vivo* stimulation with LPS. Interestingly, oral intake of yWGP counteracted LPS-induced tolerance and restored TNF-α release in peritoneal cells (Fig. 6A). However, the intake of yWGP did not significantly affect LPS-induced tolerance in cells from the spleen (Fig. 6B), bone marrow (Fig. 6C and D), or whole blood (Fig. 6E).

### 3.6 Oral exposure to yWGP does not counteract cross-tolerance in murine monocyte- and macrophage-containing cell populations

**Fig. 7:**
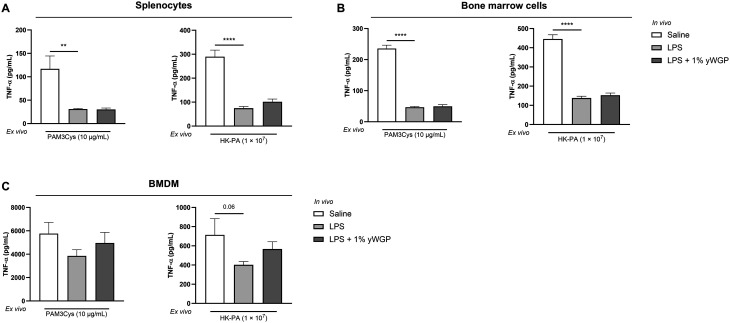
yWGP did not affect LPS-induced cross-tolerance in splenocytes and bone marrow cells. After oral exposure to a 1% w/w yWGP-supplemented diet, animals were i.p. injected with LPS. Splenocytes (A), bone marrow cells (B), and BMDMs (C) were subsequently *ex vivo* stimulated with PAM3Cys and HK-PA. Release of TNF-α was measured in supernatant collected 24 h after *ex vivo* stimulation. Data are presented as mean ± SEM, *n* = 7 for control and LPS-tolerized animals and *n* = 6 for yWGP-exposed animals. **P < 0.01, ****P < 0.0001, as analyzed with one-way ANOVA followed by Dunnett's multiple comparisons test.

To investigate whether yWGP can counteract cross-tolerance, splenocytes and bone marrow cells from yWGP-supplemented mice exposed *in vivo* to LPS were stimulated *ex vivo* with PAM3Cys or HK-PA. As observed before, levels of TNF-α and IL-6 after *ex vivo* PAM3Cys or HK-PA stimulation were significantly reduced in splenocytes collected 24 h after the *in vivo* LPS challenge (Fig. 7A and Fig. S2A†). Similar effects were observed in bone marrow cells (Fig. 7B and Fig. S2B†). In BMDMs obtained from mice *in vivo* exposed to LPS, IL-6 secretion decreased significantly following HK-PA stimulation (Fig. S2C†). For TNF-α, the reduction did not reach statistical significance (Fig. 7C), yet the trend (*p* = 0.06) was similar to that of the first study. Again, no significant effect on cytokine production was observed after stimulation with PAM3Cys (Fig. 7C and Fig. S2C†). Exposure to yWGP did not counteract cross-tolerance to PAM3Cys or HK-PA in splenocytes, bone marrow cells, or BMDMs (Fig. 7 and Fig. S2†).

## 4. Discussion

Our study demonstrates that β-glucan administered orally has the potential to counteract immunosuppression resulting from prior administration of LPS. By utilizing a combination of *in vivo* administration of LPS and yWGP, along with the *ex vivo* assessment of the secondary immune response, we found that these effects are selective for specific cell populations. This strategy of measurement reflects a situation relevant to humans and underlies a commonly observed secondary severe infection following an initial assault on the immune system.

Research investigating LPS-induced immunological tolerance often makes use of two consecutive *in vivo* LPS challenges. Consequently, these models can only assess systemic effects. Moreover, the second LPS challenge in two-hit *in vivo* animal models typically involves an otherwise lethal dose, aiming to demonstrate the development of tolerance to LPS.^21^ Implementing strategies to counteract LPS tolerance in such animal models could lead to increased mortality, which is ethically undesirable. Another widely used model to assess LPS tolerance involves evaluating cytokine production from *ex vivo*-stimulated leukocytes isolated from critically ill patients or after induction of experimental endotoxemia.^16,22^ However, since blood is the only easily accessible immune compartment in humans, this model is also limited because it only allows for the assessment of systemic effects. This is of importance as we have previously shown that effects elicited *via* immunostimulatory dietary fibers are compartment-dependent.^20^

The model used in the present study allows for a more comprehensive assessment of immunosuppression, extending beyond circulating immune cells, and for evaluation of the effect of an intervention aiming to reverse LPS (cross-) tolerance and increase immune fitness. Due to limited knowledge regarding the specific *in vivo* LPS dose required to induce *ex vivo* LPS tolerance in immune cells derived from different compartments, we commenced with a study examining various *in vivo* LPS concentrations (20, 200, 2000 μg kg^−1^). Our findings show that concentrations of 200 and 2000 μg kg^−1^ induced *ex vivo* LPS tolerance in peritoneal cells, splenocytes, bone marrow cells, BMDMs, and whole blood cultures. The reduced responsiveness observed in monocyte- and macrophage-containing cell populations was not limited to LPS, but extended to heterologous ligands (PAM3Cys and HK-PA), indicating cross-tolerance. Considering the negative impact on animal welfare observed following administration of 2000 μg kg^−1^ LPS, a dose of around 200 μg kg^−1^ LPS could serve as starting point for future studies investigating LPS-induced immunological tolerance. In a subsequent study, we demonstrated that dietary intake of 1% w/w yWGP before inducing tolerance effectively restores cytokine production by peritoneal cells. However, this restorative effect on cytokine production was not observed in other cell populations containing monocytes and macrophages.

A single sublethal dose of LPS is widely used to induce acute systemic inflammation, with plasma cytokine levels serving as primary readout parameter.^23–25^ As expected, our study demonstrated a significant increase in pro-inflammatory cytokines (TNF-α, IL-6, MCP-1) after i.p. LPS administration, peaking at 2 hours and (nearly) returning to baseline within 24 hours. This increase was accompanied by elevated anti-inflammatory IL-10 levels. The observed kinetics of plasma pro- and anti-inflammatory cytokines are in accordance with results obtained in previous research conducted in both mice^26,27^ and human subjects.^28^

Subsequently, we assessed the optimal *in vivo* LPS dose for inducing *ex vivo* LPS tolerance in immune cells. Given the crucial role of macrophages in the body's defense against infections,^29,30^ we focused on monocyte- and macrophage-containing cell populations. Cells derived from the peritoneal cavity, spleen, bone marrow, and blood of LPS-administered animals released significantly lower amounts of TNF-α and IL-6 compared to cells from PBS-treated animals upon *ex vivo* LPS stimulation. Interestingly, BMDMs from LPS-administered animals, obtained after one week of *ex vivo* culture, still showed reduced levels of TNF-α and IL-6 upon stimulation. This suggests that the tolerogenic state persists in these cells for at least a week, which has been previously demonstrated in splenic dendritic cells.^31^ As far as we know this is the first time this phenomenon is reported for BMDMs. The most pronounced *ex vivo* LPS tolerance response was consistently observed across all organs at doses of 200 and 2000 μg kg^−1^ LPS. Due to the compromised welfare of animals administered with 2000 μg kg^−1^ LPS, we selected 200 μg kg^−1^ for subsequent experiments. Notably, this LPS dose falls within the range of doses previously investigated in the limited studies analyzing *ex vivo* LPS tolerance in mice (30–1000 μg kg^−1^).^32–34^

Cross-tolerance is a phenomenon in which primary activation of one PRR, for example, LPS activation of TLR4, results in non-responsiveness to secondary stimuli that activate other PRRs (*e.g.*, TLR2 or TLR5). In the present study, we demonstrated that *in vivo* LPS administration resulted in a suppressed release of pro-inflammatory cytokines upon *ex vivo* stimulation with PAM3Cys and HK-PA. These results confirm recent findings regarding cross-tolerance to components of both Gram-positive (lipoteichoic acid) and Gram-negative bacteria (LPS).^35,36^ Notably, profound tolerance and cross-tolerance to LPS were established within 24 hours, as also reported in humans,^37^ and possibly contribute to increased susceptibility to secondary infections leading to higher morbidity and mortality in critically ill patients.^38^

Reducing immunosuppression in order to clear the primary infection or to prevent secondary infections is of utmost importance. Restoring inflammation-induced immunosuppression *via* immune-fitness-enhancing strategies has gained attention due to the limited efficacy of anti-inflammatory treatments and the high mortality associated with secondary hospital infections. Recently, studies have shown that recombinant IFNγ can partially restore metabolic function in tolerized monocytes from patients with sepsis.^12^ This finding highlights the potential of using innate immune “trainers” to mitigate tolerance as a therapeutic approach. We hypothesized that the β-glucan yWGP could counteract LPS-induced tolerance, given its immunomodulatory effects.^15,39,40^ Previous studies have demonstrated that *in vitro* exposure to β-glucan can reverse tolerance in primary macrophages,^15,16^ and recently, we found that dietary intake of yWGP leads to enhanced innate immune responsiveness in mouse blood and bone marrow compartments.^20^

In the current study, we therefore investigated whether dietary intake of yWGP could also rescue macrophages from LPS-induced immunosuppression. To this end, we examined the production of TNF-α and IL-6 by various murine monocyte- and macrophage-containing cell populations upon an *ex vivo* LPS challenge. While yWGP intake could prevent LPS tolerance in peritoneal cells, this effect was not observed in splenocytes, bone marrow cells, and whole blood cultures. A possible explanation for this discrepancy could be that peritoneal cells comprise a population that includes a considerable proportion of highly differentiated or specialized tissue macrophages.^41^ These cells possess the capacity to release significant quantities of both pro-inflammatory and anti-inflammatory cytokines and are highly phagocytic. An alternative explanation for the impact being limited to peritoneal cells may be the localization of LPS administration within the peritoneal region and the proximity of these peritoneal cells to the intestinal lymphoid tissue, where oral β-glucans exert their effects.^42,43^ These findings emphasize the need to investigate the uptake and migration of yWGP, resulting in either direct or indirect effects on various immunological tissues. Future research should also assess the timing of yWGP administration. Successful reversal of LPS tolerance was previously achieved by administering recombinant IFNγ between the two LPS challenges.^44^ However, it is important to note that there is a limited time frame for intervention, as previous research has shown that *ex vivo* LPS tolerance in whole blood and spleen resolves within 1 week.^31,45^ In addition to examining the effects of yWGP on LPS-induced immunosuppression, we also assessed its influence on plasma cytokine levels following i.p. LPS administration. However, no significant effect of yWGP was observed in this regard. Male mice were used in this study and it is known that they are more sensitive to LPS than females. In addition, we aimed to avoid the potential influence of hormonal cycle variations on inflammatory responses. Therefore, further research including both male and female animals is essential to confirm the present findings and to account for sex difference when extrapolating these results to clinical studies.

In conclusion, this study demonstrates that an i.p. LPS dose of 200 μg kg^−1^ induces *ex vivo* immunosuppression in various monocyte- and macrophage-containing cell populations (*i.e.*, peritoneal cells, splenocytes, bone marrow cells, BMDMs, and whole blood cultures). Interestingly, dietary intake of 1% w/w yWGP for 2 weeks prior to LPS administration effectively counteracted the development of LPS tolerance in peritoneal cells. At the same time, restorative effects on cytokine production in other monocyte- and macrophage-containing cell populations were not observed. Future research should focus on timing of yWGP administration, mechanisms of intestinal absorption and systemic distribution. The current investigation provides preliminary insights into the potential of a nutritional strategy to reduce immunosuppression, which represents a relevant clinical challenge that is commonly observed in vulnerable, immunocompromised patient populations.

## Author contributions

B.G.J.M., J.J.M., J.v.B., C.G., M.v.D., K.v.N., and S.A. conceived of and designed the study. B.G.J.M. and S.A. performed the experiments. B.G.J.M. and S.A. analyzed the data and wrote the manuscript, which was reviewed by all co-authors.

## Data availability

The data supporting this article have been included as part of the ESI.†

## Conflicts of interest

J.v.B. and M.v.D. are employed by Danone Nutricia Research.

The remaining authors declare that the research was conducted in the absence of any commercial or financial relationships that could be construed as a potential conflict of interest.

## Supplementary Material

FO-016-D4FO05223D-s001

FO-016-D4FO05223D-s002

FO-016-D4FO05223D-s003

FO-016-D4FO05223D-s004
