# Supplementary figures and images for: Induction of endotoxin tolerance in murine monocyte and macrophage cell populations – optimal LPS dose and compartment-specific reversal by β-glucan
Source: Food Funct. 2025 Feb 7;16(4):1576–87. doi: 10.1039/d4fo05223d (PMC11803501; doi:10.1039/d4fo05223d)

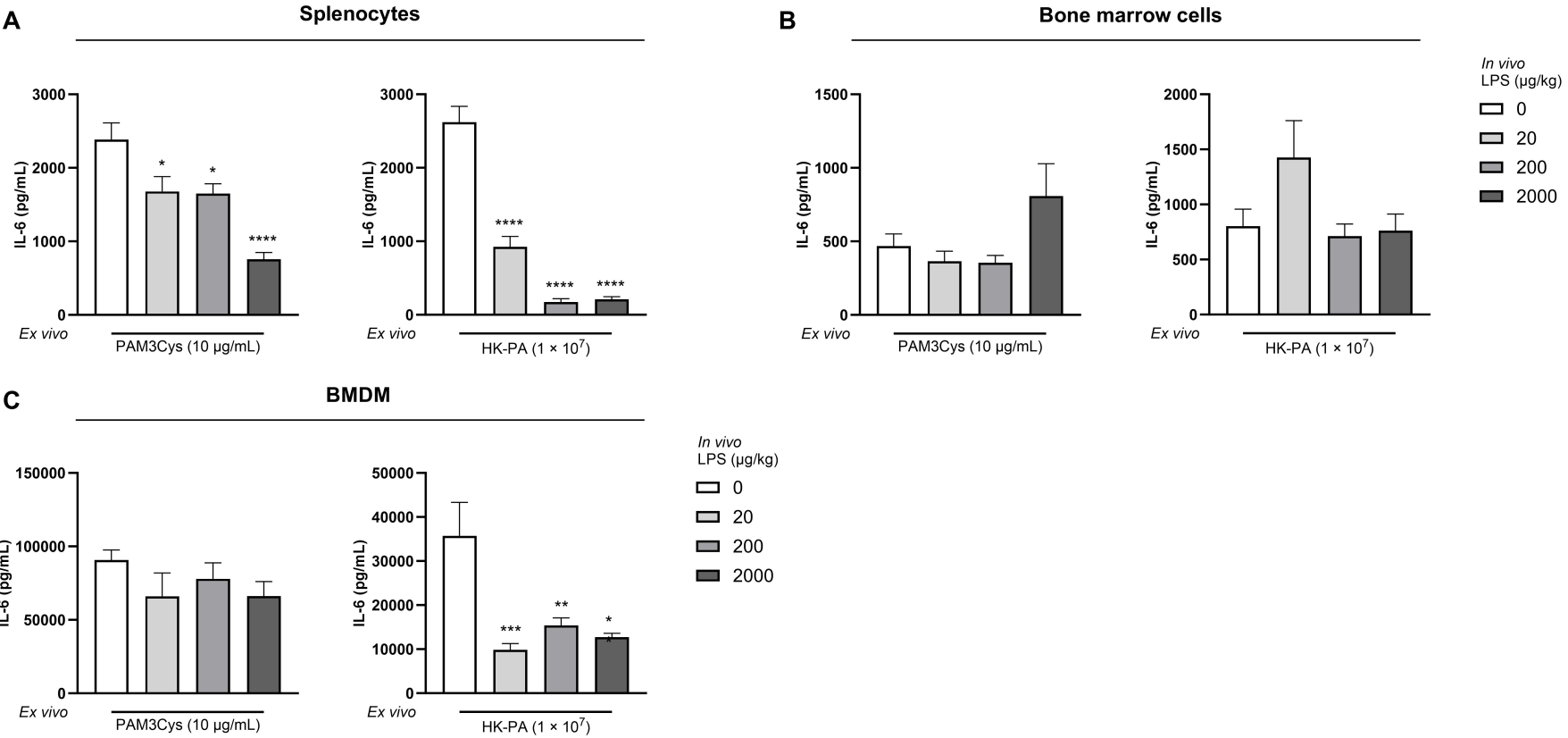

Supplement: FO-016-D4FO05223D-s001 [file FO-016-D4FO05223D-s001.pdf]

**A**

**Splenocytes**

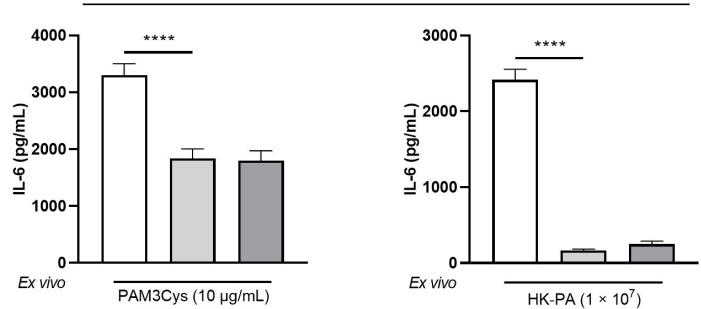

**B**

**Bone marrow cells**

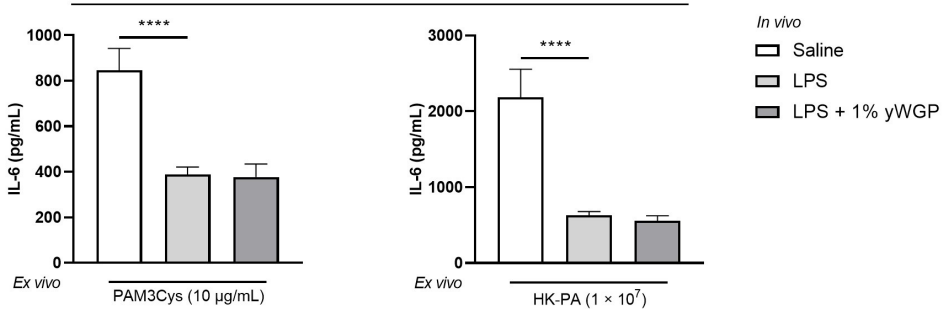

**C**

**BMDM**

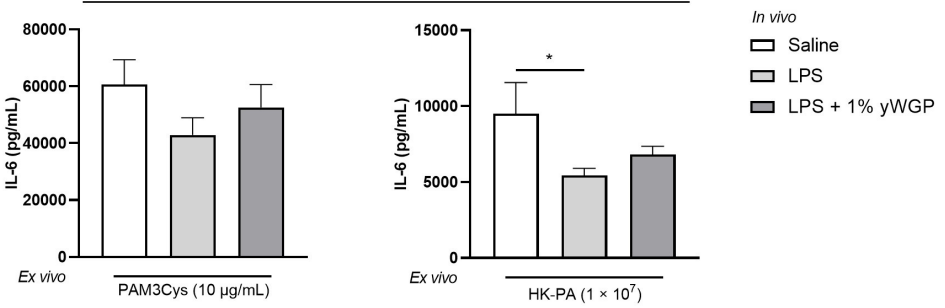

Supplement: FO-016-D4FO05223D-s002 [file FO-016-D4FO05223D-s002.pdf]
