# Supplementary material for: Induction of endotoxin tolerance in murine monocyte and macrophage cell populations – optimal LPS dose and compartment-specific reversal by β-glucan
Source: Food Funct. 2025 Feb 7;16(4):1576–87. doi: 10.1039/d4fo05223d (PMC11803501; doi:10.1039/d4fo05223d)
Supplement: FO-016-D4FO05223D-s003 [file FO-016-D4FO05223D-s003.pdf]

This journal is © The Royal Society of Chemistry 2025

Supplementary Figure legends

Figure S1. *In vivo* exposure to LPS induced *ex vivo* cross-tolerance to PAM3Cys and HK-PA. IL-6 concentrations measured in supernatant after *ex vivo* PAM3Cys and HK-PA stimulation of splenocytes (**A**), bone marrow cells (**B**), and BMDMs (**C**) from mice *in vivo* exposed to LPS (20, 200, and 2000 μg/kg) or PBS. Data are presented as mean ± SEM, n = 5/group. *$P < 0.05$, **$P < 0.01$, ***$P < 0.001$, ****$P < 0.0001$, as analyzed with one-way ANOVA followed by Dunnett's multiple comparisons test.

Figure S2. No effect of yWGP on LPS-induced cross-tolerance in spleen- and bone marrow-derived cells. yWGP-exposed animals were *i.p.* injected with LPS. IL-6 release was subsequently measured in the supernatant of splenocytes (**A**), bone marrow cells (**B**), and BMDMs (**C**) *ex vivo* stimulated with PAM3Cys and HK-PA for 24 h. Data are presented as mean ± SEM, n = 7 for control and LPS-tolerized animals and n = 6 for yWGP-exposed animals. *$P < 0.05$, ****$P < 0.0001$, as analyzed with one-way ANOVA followed by Dunnett's multiple comparisons test (**A, B**) or Kruskal-Wallis test followed by Dunn's multiple comparisons test (**C**).
