# Supplementary material for: Induction of endotoxin tolerance in murine monocyte and macrophage cell populations – optimal LPS dose and compartment-specific reversal by β-glucan
Source: Food Funct. 2025 Feb 7;16(4):1576–87. doi: 10.1039/d4fo05223d (PMC11803501; doi:10.1039/d4fo05223d)
Supplement: FO-016-D4FO05223D-s004 [file FO-016-D4FO05223D-s004.pdf]

Supplementary Information (SI) for Food & Function.
This journal is © The Royal Society of Chemistry 2025

Table S1. Composition of the intervention diets.

Ingredients (*g/kg dry matter*) \ Diet name	Control	WGP
		1%
**Carbohydrates**		
Cornstarch	452.9	418.6
Dextrinized cornstarch	151.3	174.8
Sucrose	97.3	97.3
**Protein**		
Casein	140.0	139.5
**Fat**		
Soybean oil	38.6	35.7
**Fibers**		
Cellulose	47.5	38.0
yWGP	0.0	12.7
**Others**		
Mineral mix[1]	35.0	35.0
Vitamin mix[1]	10.0	10.0
Choline bitartrate	2.5	2.5
Tert-butylhydroquinone	0.008	0.008

The control diet is AIN-93M without the antioxidant L-cysteine added. yWGP (yeast-derived whole β-glucan particle) was added to the AIN-93M diet (with a correction for fiber purity; 75%) at the expense of cellulose. To be isocaloric to the control diet, the fiber diet needed to be adjusted in added carbohydrates and protein. Since sucrose might influence intestinal transit, it was kept equal for all diets.

Table S2. Physicochemical composition of yWGP.

Preparation	Relative Solubility (%)	Monosaccharides (mol%)							Total saccharide content (w/w%)	Protein content (%)	LPS (EU/ mg)
		Rha	Ara	Xyl	Man	Gal	Glc	Uronic acid			
yWGP	< 13	0	1	0	0	0	96	3	64	16	N.D.

Rha, rhamnose; *ara*, arabinose; *xyl*, xylose; *man,* mannose; *gal*, galactose; *glc*, glucose; *LPS*, lipopolysaccharide; *N.D.*, not detectable. The physicochemical composition data of yWGP is derived from [15].
